# Automated measurement of fetal head circumference using 2D ultrasound images

**DOI:** 10.1371/journal.pone.0200412

**Published:** 2018-08-23

**Authors:** Thomas L. A. van den Heuvel, Dagmar de Bruijn, Chris L. de Korte, Bram van Ginneken

**Affiliations:** 1 Diagnostic Image Analysis Group, Department of Radiology and Nuclear Medicine, Radboud University Medical Center, Nijmegen, the Netherlands; 2 Medical Ultrasound Imaging Center, Department of Radiology and Nuclear Medicine, Radboud University Medical Center, Nijmegen, the Netherlands; 3 Department of Obstetrics and Gynecology, Radboud University Medical Center, Nijmegen, the Netherlands; 4 Fraunhofer MEVIS, Bremen, Germany; City University London, UNITED KINGDOM

## Abstract

In this paper we present a computer aided detection (CAD) system for automated measurement of the fetal head circumference (HC) in 2D ultrasound images for all trimesters of the pregnancy. The HC can be used to estimate the gestational age and monitor growth of the fetus. Automated HC assessment could be valuable in developing countries, where there is a severe shortage of trained sonographers. The CAD system consists of two steps: First, Haar-like features were computed from the ultrasound images to train a random forest classifier to locate the fetal skull. Secondly, the HC was extracted using Hough transform, dynamic programming and an ellipse fit. The CAD system was trained on 999 images and validated on an independent test set of 335 images from all trimesters. The test set was manually annotated by an experienced sonographer and a medical researcher. The reference gestational age (GA) was estimated using the crown-rump length measurement (CRL). The mean difference between the reference GA and the GA estimated by the experienced sonographer was 0.8 ± 2.6, −0.0 ± 4.6 and 1.9 ± 11.0 days for the first, second and third trimester, respectively. The mean difference between the reference GA and the GA estimated by the medical researcher was 1.6 ± 2.7, 2.0 ± 4.8 and 3.9 ± 13.7 days. The mean difference between the reference GA and the GA estimated by the CAD system was 0.6 ± 4.3, 0.4 ± 4.7 and 2.5 ± 12.4 days. The results show that the CAD system performs comparable to an experienced sonographer. The presented system shows similar or superior results compared to systems published in literature. This is the first automated system for HC assessment evaluated on a large test set which contained data of all trimesters of the pregnancy.

## Introduction

Ultrasound imaging is widely used for screening and monitoring of pregnant women, since it is a low-cost, real-time and non-invasive imaging method. However, acquisition of ultrasound images is operator-dependent and the images are characterized by attenuation and speckle and may contain artifacts such as shadows and reverberations, making their interpretation complex. During the ultrasound screening examination, biometric measurements of the fetus such as the crown-rump length (CRL) and the head circumference (HC) are often computed to determine the gestational age (GA) and to monitor growth of the fetus. The CRL is the most accurate measurement for estimating the GA of the fetus between 8 weeks and 4 days (commonly noted as: 8^+4^ weeks) and 12^+6^ weeks. After 13 weeks, the HC is used the most accurate measurement to determine the GA, because it is not possible to accurately measure the CRL anymore. The guidelines state that HC should be measured in a transverse section of the head with a central midline echo, interrupted in the anterior third by the cavity of the septum pellucidum with the anterior and posterior horns of the lateral ventricles in view [1]. The biometric measurements are obtained manually, which leads to inter- and intra-observer variability. An accurate automated system could reduce measuring time and variability, because it does not suffer from intra-observer variability. Worldwide, 99% of all maternal deaths occur in developing countries. Skilled care before, during and after childbirth can save the lives of women and newborn babies [2]. Unfortunately, there is still a severe shortage of well-trained sonographers in low resource settings. This keeps ultrasound screening out of reach for most pregnant women in these countries [3]. An automated system could assist inexperienced human observers in obtaining an accurate measurement. In this work, we focus on measuring the HC because this measurement can be used to determine the GA and monitor growth of the fetus. In addition, the fetal head is more easily detectable compared to the fetal abdomen.

Systems for automatic HC measurement have been presented using randomized Hough transform [4, 5], Haar-Like features [6–9], multilevel thresholding [10], circular shortest paths [11], boundary fragment models [12], semi-supervised patch based graphs [13], active contouring [14, 15], intensity based features [16] and texton based features [17]. Although these methods show promising results, they were evaluated on a relatively small amount of data (10 to 175 test images). Furthermore, none of these papers used images of fetuses from all trimesters of pregnancy. We present a system that was developed using 999 ultrasound images and evaluated on a large independent test set of 335 ultrasound images from all trimesters. The presented quantification system was designed to be as fast and robust as possible and the results were compared to the methods presented in literature. A complete overview of the comparison between our method and previous publications is presented in Section Comparison to literature.

## Materials and methods

### Data

A total of 1334 two-dimensional (2D) ultrasound images of the HC were collected from the database of the Department of Obstetrics of the Radboud University Medical Center, Nijmegen, the Netherlands. The ultrasound images were acquired from 551 pregnant women who received a routine ultrasound screening exam between May 2014 and May 2015. Only fetuses that did not exhibit any growth abnormalities were included in this study. Images were acquired by experienced sonographers using either the Voluson E8 or the Voluson 730 ultrasound device (General Electric, Austria). The local ethics committee (CMO Arnhem-Nijmegen) approved the collection and use of this data for this study. Due to the retrospective data collection, informed consent was waived. All data was anonymized according to the tenets of the Declaration of Helsinki.

**Fig 1:**
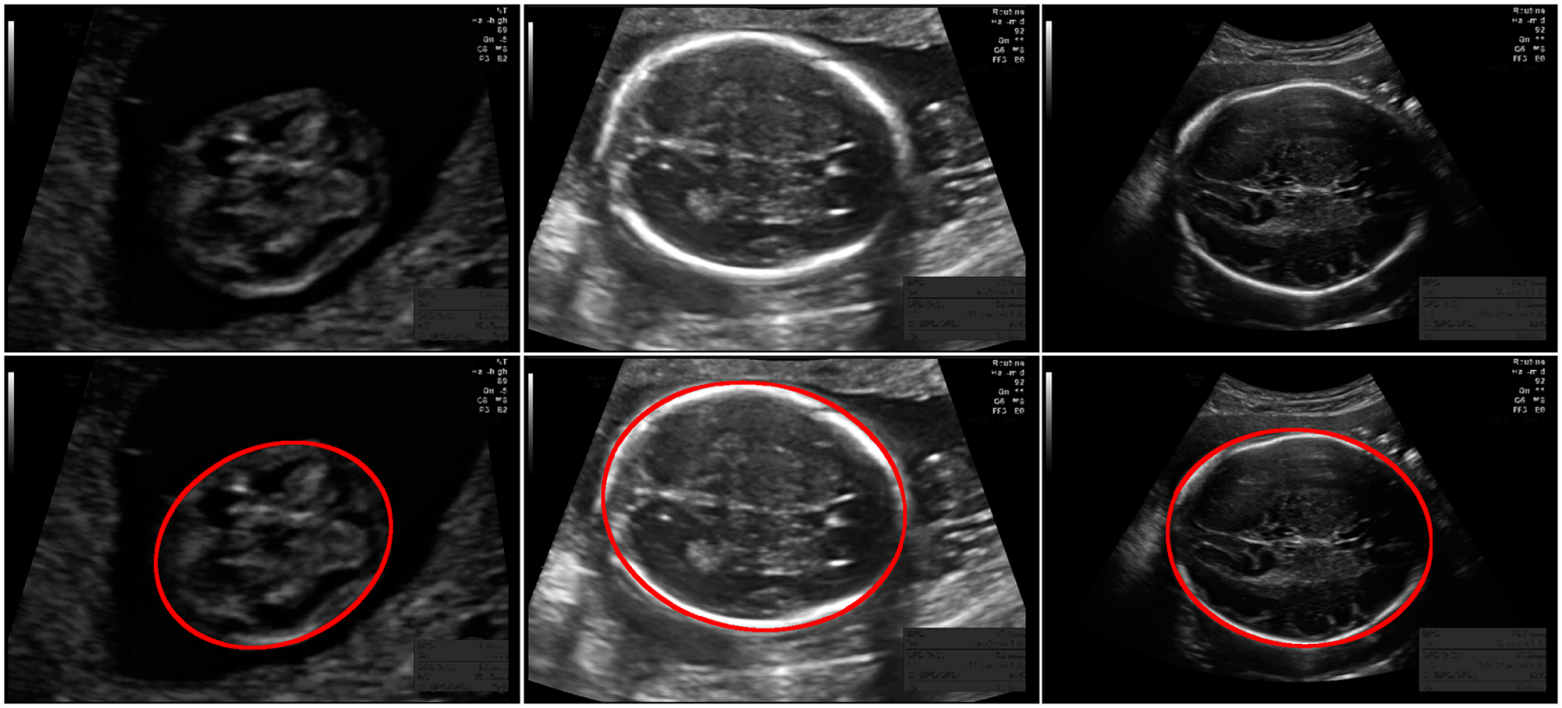
Example ultrasound images. From top to bottom: without annotation and with annotation in red. From left to right: first trimester with an HC of 65.1 mm (pixel size of 0.06 mm), second trimester with an HC of 167.9 mm (pixel size of 0.12 mm) and third trimester with an HC of 278.4 mm (pixel size of 0.24 mm). Note that the skull is not yet visible as a bright structure in the first trimester.

**Fig 2:**
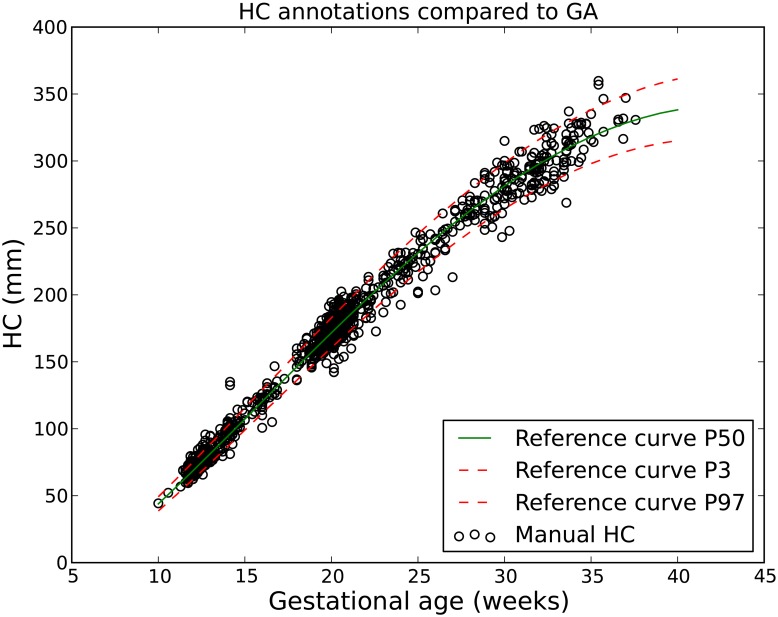
Distribution of HC and GA for the study data. The x-axis represents the GA that was estimated using the CRL. The y-axis represents the HC measured by the experienced sonographer.

The size of each 2D ultrasound image was 800 by 540 pixels with a pixel size ranging from 0.052 to 0.326 mm. This large variation in pixel size is a result of adjustments in the ultrasound settings by the sonographer (depth settings and amount of zoom are routinely varied during the examination) to account for the different sizes of the fetuses. Fig 1 shows example ultrasound images from each trimester. The distribution of the GA in this study is shown in Fig 2. Most data were acquired after 12 and 20 weeks of pregnancy, since these are standard time points of routine ultrasound screening for pregnant women in the Netherlands. During each exam, the sonographer manually annotated the HC. This was done by drawing an ellipse that best fits the circumference of the head. Fig 2 also shows the comparison between the distribution of the HC and the growth curve of Verburg *et al*. [1]. The reference GA was determined with a CRL measurement between 20 mm (8^+4^ weeks) and 68 mm (12^+6^ weeks). All the HCs that fell outside the 3-97 percent confidence interval of the curve of Verburg *et al*. [1] were individually checked to ensure no mistakes were made during data collection.

The data was randomly divided into a training set and a test set of 75 percent and 25 percent, respectively. The GAs were proportionally balanced between the data sets as shown in Table 1. All images that were made during one echographic examination were assigned to either the training or the test set. An independent data set of HC annotations of the images in the test set was created by TLAvdH, a medical researcher who has a technical background in ultrasound imaging and received training by an experienced sonographer in measuring the HC.

**Table 1:** Number of images in the training and the test set.

Trimester	Training set	Test set
First	165	55
Second	693	233
Third	141	47
**Total**	**999**	**335**

### Quantification system

**Fig 3:**
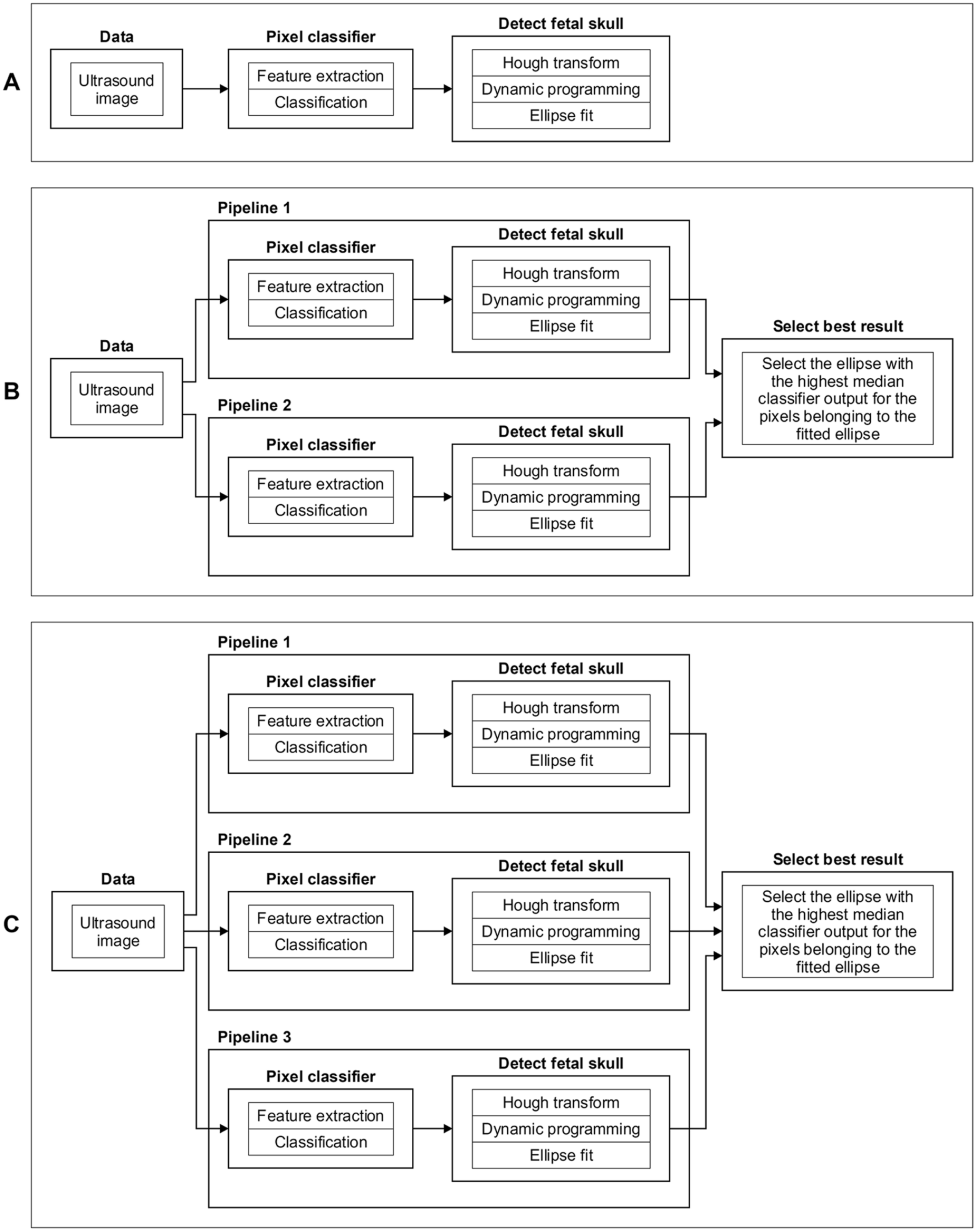
Overview of the three evaluated quantification systems *A*, *B*, and *C*. System *A* was optimized on training data from all trimesters. System *B* has two pipelines: pipeline 1 was optimized on training data from trimester one and pipeline 2 was optimized on training data from trimester two and three. System *C* uses three pipelines: pipeline 1, 2 and 3 were optimized on training data from trimester one, two and three, respectively. All pipelines of a quantification system are computed when the HC is measured in a test ultrasound image.

In this study, three variations of the quantification system, indicated as system *A*, *B*, or *C*, were optimized and evaluated to investigate the influence of the changing appearance of the fetal head during pregnancy on the performance of the system. An overview of the three systems is shown in Fig 3. All three systems contain the same two steps: First, Haar-like features were computed from the ultrasound images to train a random forest classifier (RFC) to locate the fetal skull. Next, the HC was extracted using Hough transform, dynamic programming and an ellipse fit. Both steps are described in detail in the following subsections. System *A* uses one pipeline that was optimized on training data from all trimesters. It can be seen in Fig 1 that the fetal skull is not clearly visible in the first trimester. To deal with this different appearance, system *B* uses two pipelines to measure the HC: one pipeline was optimized on training data from the first trimester and the other pipeline was optimized on training data from the second and third trimesters. System *C* uses three pipelines, which were optimized on training data from the first, second and third trimester separately. In a low-resource setting the trimester of the fetus is commonly unknown. For systems with multiple pipelines, a selection method was used to automatically select the best fitted ellipse. This allows the system to automatically measure the HC without requiring the trimester to be known in advance.

#### Pixel classifier

The first step of the three quantification systems consists of a pixel classifier that emphasizes the fetal skull and reduces artifacts in the ultrasound image, by computing the likelihood that each pixel in the image has of being part of the fetal skull. This makes the detection of the fetal skull in the second step more robust.

**Fig 4:**
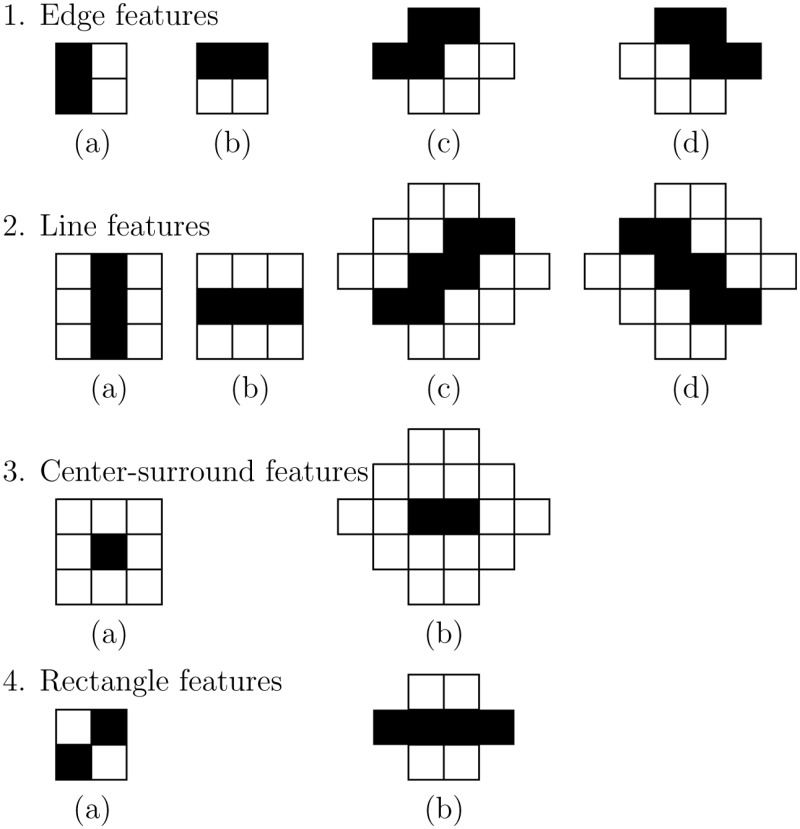
Overview of the twelve Haar-like features utilized in the quantification system. From top to bottom: 1. Edge features in horizontal and vertical direction (kernel size of two by two pixels). 2. Line features in horizontal en vertical direction (kernel size of three by three pixels). 3. Center-surround features (kernel size of three by three pixels). 4. Rectangle features (kernel size of two by two pixels). The left side of each row represents the features in upright direction. The right side of each row represents the features in rotated direction. The height and width of the features in rotated direction are larger compared to the upright direction, but they capture the same relationship between the neighboring pixels.

**Feature extraction:** Haar-like features [18] were used to be able to discriminate between background pixels and pixels that belong to the fetal skull. Viola and Jones [19] have shown that using an integral image enables the rapid computation of these features. Fig 4 shows the twelve different Haar-like features that were used for the pixel classification. The Haar-like features in rotated direction have a larger kernel width and height compared to the upright direction, but they capture the same relationship between the neighboring pixels. The Haar-like features were computed in different kernel sizes. To make these kernels invariant to the pixels size of the ultrasound image, all features were computed in millimeters. The pixel size of each Haar-like feature was chosen as close to the millimeter scale as possible. As a consequence, the kernel size of the Haar-like features increases when the pixel size of an ultrasound image decreases. A larger kernel size will result in a higher kernel response. To make the response of the feature independent from its kernel size, the Haar-like features were normalized. Normalization was performed by dividing the positive and negative coefficients of the kernel by their respective areas.

**Classification:** An OpenCV implementation of the RFC [20] was used for pixel classification. Positive samples were obtained from pixels annotated by the sonographers as the HC. The same number of negative samples were obtained from pixels randomly taken from the background with a minimal distance *d*_*min*_ from the annotation. When negative samples were obtained too close to the annotation they resemble positive samples, since the manually drawn ellipse will never fit the outer edge of the skull perfectly. This problem was solved by increasing *d*_*min*_, which was optimized within the training set. Data augmentation was applied by flipping the ultrasound image horizontally, which resembles an acquisition with a flipped ultrasound transducer. The pixel classifier produces a likelihood map with a per pixel estimate of being part of the fetal skull. This likelihood map was visualized with a color map ranging from green to red, where a high likelihood was shown in red.

#### Detect fetal skull

The likelihood map of the pixel classifier was used to detect the fetal skull in three steps. First, a Hough transform was applied to detect the center of the fetal skull. Secondly, dynamic programming was used to detect the outside of the fetal skull. Finally, an ellipse was fitted on the result of the dynamic programming algorithm to measure the HC.

**Hough transform:** An itk implementation of the Hough transform algorithm [21] was used to detect the center of the fetal skull from the likelihood map of the pixel classifier. Every classification pipeline has a GA ranging from the minimum GA, *GA*_*min*_, to the maximum GA, *GA*_*max*_. The minimum radius, *r*_*min*_, of each classification pipeline was set to the half of the biparietal diameter (BPD) of the *GA*_*min*_ on the P3 curve of Verburg *et al*. [1]. The maximum radius, *r*_*max*_, of each classification pipeline was computed using Eq (1) in which the HC and BPD are taken from the *GA*_*max*_ of the P97 curve of Verburg *et al*. [1]. The Hough transform was not used to measure the HC because the fitted circle will not give a good estimation of the elliptical shape of the fetal skull. Instead, the detected center was used for initialization of the dynamic programming algorithm (as explained in the next step), which is computational more efficient than fitting an ellipse using Hough transform.

$$\begin{array}{c} r_{max}=\lceil \frac{HC}{\pi }-\frac{BPD}{2}\rceil \end{array}$$ (1)

**Fig 5:**
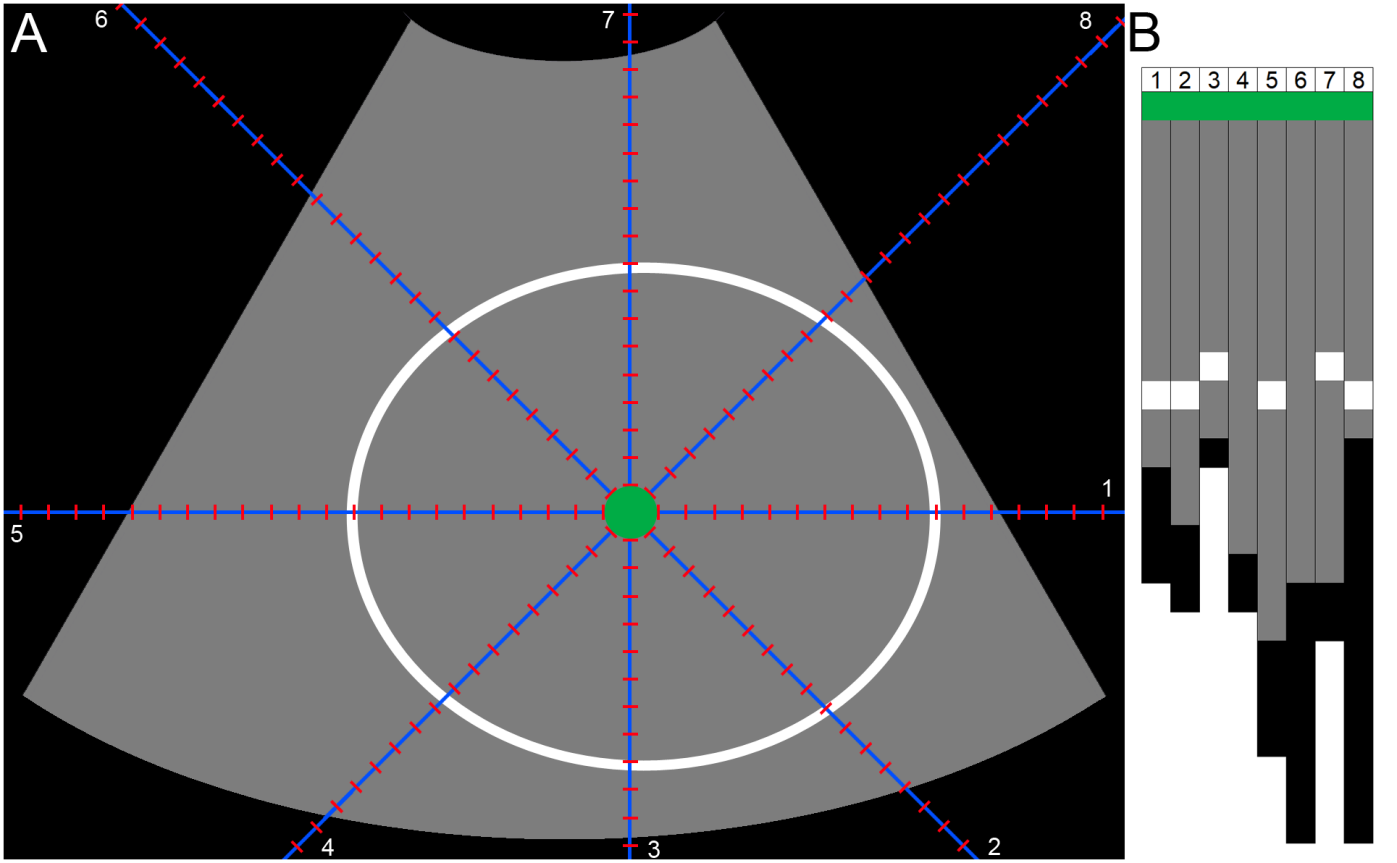
A: Perfect pixel classifier likelihood map where only the fetal skull has a high probability (depicted in white) and the background a low probability (depicted in gray). The pixels outside of the FOV are depicted in black. The center detected by the hough transform is depicted in purple and the radial offset is depicted in green. This schematic example uses eight angles (*N*_*angles*_) for the polar transform (depicted in blue). The sampling distance (*S*_*dis*_) is depicted in red. B: The output of the polar transform. The dynamic programming algorithm is used to extract the shortest path from left to right.

**Dynamic programming:** Dynamic programming was used to extract the pixels belonging to the outside of the fetal skull [22]. Dynamic programming was used, because it can be computed very efficiently compared to other methods like active contouring. Fig 5 shows a schematic example of the dynamic programming algorithm. Dynamic programming was used in a polar transform of the pixel classifier likelihood map to find the shortest path from the left to the right side of Fig 5B. The polar transform uses a preset number of angles, *N*_*angles*_, around the center point that was detected with the Hough transform algorithm. The sampling distance, *S*_*dis*_, in radial direction was increased to make the algorithm less sensitive to noise and spurious responses in the likelihood map and to a decrease computation time. When *S*_*dis*_ becomes too large, the resolution of the polar transform decreases and eventually the dynamic programming algorithm will fail to detect the fetal skull. An optimal value for *S*_*dis*_ was determined on the training set. To make the dynamic programming algorithm less sensitive to small circular structures in the likelihood map, a radial offset of 5 mm and 10 mm was taken for the second and third trimester, respectively. According to the annotation protocol for HC measurements, the HC must be detected at the outside edge of the fetal skull [1]. Although the RFC was trained with annotations that describe the outside of the fetal skull, the Haar-like features were not able to distinguish between inside and outside of the fetal skull. Therefore, the RFC detected all pixels belonging to the fetal skull instead of only those that belong to the outside of the fetal skull. For this reason, the dynamic programming algorithm detected the midline of the skull. To solve this problem, a second dynamic programming algorithm was computed in the polar transform of the ultrasound image. This algorithm uses the same center and number of angles, *N*_*angles*_, as the first dynamic programming algorithm, but without any downsampling in radial direction to maintain detailed information about the edge of the skull. To detect the outside of the fetal skull, the derivative of the ultrasound image in radial direction was computed. Pilot experiments showed that the fetal skull is only a few millimeters thick. To restrict the second dynamic programming algorithm to the area that is likely to contain the fetal skull, the second dynamic programming algorithm was only computed on the area within a distance of 2 mm from the first dynamic programming result. It is not advisable to directly apply dynamic programming to the derivative of the ultrasound image in radial direction because this would be overly sensitive to noise in ultrasound image. The result of the second dynamic programming algorithm, computed on the derivative of the ultrasound image, was taken as the final result for the ellipse fit in the next step.

**Ellipse fitting:** A direct least square fitting of ellipses [23] was used to determine the HC from the extracted pixels of the dynamic programming algorithm. Only the pixels detected by the dynamic programming algorithm within the highest fifth percentile of the likelihood map of the pixel classifier were used to fit the ellipse, because these pixels have a high likelihood for being part of the fetal skull. The fitted ellipse was required to have a circumference of at least 38.6 mm. This is the smallest reported HC on the curve of Verburg *et al*. [1] and will therefore prevent the quantification system from detecting small circular structures or noise in the image.

#### Select best result

All pipelines of a quantification system were computed when the HC was measured in a test ultrasound image. In a low-resource setting the trimester of the fetus is commonly unknown, so quantification systems *B* and *C* will produce two and three fitted ellipses, respectively. To allow the system to fully automatically measure the HC, the ellipse with the highest median value of the first dynamic programming algorithm on the pixel classifier likelihood map was selected as the final result.

## Experiments

Four experiments were performed to evaluate the performance of the three quantification systems and compare them to the manual annotations of the experienced sonographer (observer 1) and the medical researcher (observer 2). First, the parameters of the pipelines were optimized for each system. Secondly, the HC measured by observer 1 was used as a reference to compare the HC measured by the three systems and the HC measured by observer 2. Thirdly, the measured HCs were used to estimate the GAs which were compared to the GAs that were estimated using the CRL (measured in the first trimester of the pregnancy). Finally, we checked for indications of overfitting.

### System parameter optimization

All parameters in the three quantification systems were optimized within the training set using a three-fold cross-validation. Optimization of five parameters was performed to improve the system performance (the parameter settings can be found in Table 2). First, the number of trees in the RFC was increased until the performance of the classifier was stable. Increasing the number of trees increases the computation time, so the lowest number of trees which showed a stable performance was used during optimization of the other parameters. Secondly, the scales of the Haar-like features were optimized. Starting with the optimum single scale, additional scales were only included when they improved the result. Thirdly, both the minimal distance, *d*_*min*_ and *S*_*dis*_ were increased until the performance did not improve anymore. Finally, the number of angles, *N*_*angles*_, used for the polar transform was decreased as long as the performance of the system did not decrease, to speed up computation time.

**Table 2:** Parameter sets for optimizing systems *A*, *B*, and *C*.

Parameter	Set
Number of trees in RFC	*N*_*trees*_∈{1, 2, 5, 10, 20, 50, 100}
Haar-like feature scales (mm)	*F*_*scales*_∈{0.1, 0.2, 0.5,…,40, 45, 50}
Background sampling (mm)	*d*_*min*_∈{0, 0.1, 0.2, 0.3}
Polar transform (mm)	*S*_*dis*_∈{0, 0.1, 0.2, 0.3, 0.4, 0.5}
Polar transform	*N*_*angles*_∈{360, 270, 180}

### HC comparison

The HC annotations of observer 1 were used as a reference to compare the performance of quantification system *A*, *B*, or *C*, as well as the observer 2 using the difference (DF), the absolute difference (ADF), the Hausdorff distance (HD) [24] and the Dice similarity coefficient (DSC) [25].

DF was defined as:

$$\begin{array}{c} DF=HC_{S}-HC_{R}, \end{array}$$ (2)

where *HC*_*R*_ is the HC measured by observer 1 and *HC*_*S*_ is the HC measured by observer 2 or quantification system *A*, *B* or *C*.

ADF was defined as:

$$\begin{array}{c} ADF=|HC_{S}-HC_{R}| \end{array}$$ (3)

HD was defined as:

$$\begin{array}{c} H(S,R)=max(h(S,R),h(S,R)), \end{array}$$ (4)

where *R* = {*r*_1_, …, *r*_*q*_} are the pixels from observer 1 and *S* = {*s*_1_, …, *s*_*p*_} are the pixels from observer 2 or quantification system *A*, *B* or *C*, given:

$$\begin{array}{c} h(S,R)=\max_{s\epsilon S}\max_{r\epsilon R}∥s-r∥. \end{array}$$ (5)

DSC was defined as:

$$\begin{array}{c} DSC=\frac{2\cdot |Area_{S}\cap Area_{R}|}{|Area_{S}|+|Area_{R}|}, \end{array}$$ (6)

where *Area*_*R*_ is the area of the annotation of observer 1 and *Area*_*S*_ is the area of the annotation of observer 2 or the quantification system *A*, *B* or *C*.

Statistical analysis was performed to determine whether the difference was significant (*p* < 0.05). When the tested data was normally distributed according to the Shapiro-Wilk test, a paired T-Test was performed using SPSS (version 20.0). Otherwise, a Wilcoxon Signed Rank Test was performed. Although not all distributions were normally distributed, the tables in the Results Section show the mean and standard deviation, because this makes a comparison with values provided in previous literature possible.

### GA comparison

The GA from the HC of the quantification systems and the observers was estimated using the P50 curve from Verburg *et al*. [1]. The reference GA was determined with a CRL measurement between 20 mm (8^+4^ weeks) and 68 mm (12^+6^ weeks). The differences between the estimated GA and the reference GA were computed for evaluation of the results. The same statistical tests as explained in the previous Section were used to determine whether the difference in GA was significant.

### Overfitting

The best performing quantification system was evaluated on the training data to investigate whether overfitting of the system parameters had occurred.

## Results

### System parameter optimization

Table 3 shows the final parameter settings of the three quantification systems, as determined by running the optimization procedure on the training set explained in the Experiments Section. The Haar-like feature scales are sorted by importance. Note that both the most important Haar-like feature scale, *F*_*scales*_, and the downsampling of the dynamic programming, *S*_*dis*_, increases with the trimester.

**Table 3:** Final parameter settings of quantification systems *A*, *B*, and *C* after parameter optimization.

	System A	System B		System C		
Computed on trimester(s)	1, 2 and 3	1	2 and 3	1	2	3
Number of trees RFC, *N*_*trees*_	10	10	10	10	10	10
Haar-Like feature scales, *F*_*scales*_(mm)	6, 20	2.5, 0.5, 11	7,11	2.5, 0.5, 11	7	9,12
Background sampling, *d*_*min*_ (mm)	0	0.2	0.2	0.2	0.1	0.1
Hough transform, *r*_*min*_ (mm)	5	5	12	5	12	34
Hough transform, *r*_*max*_ (mm)	61	18	61	18	50	61
Polar transform, *S*_*dis*_ (mm)	0.4	0.2	0.4	0.2	0.3	0.5
Polar transform, *N*_*angles*_	270	270	270	270	270	270

### Visualization of computation steps of quantification system C

**Fig 6:**
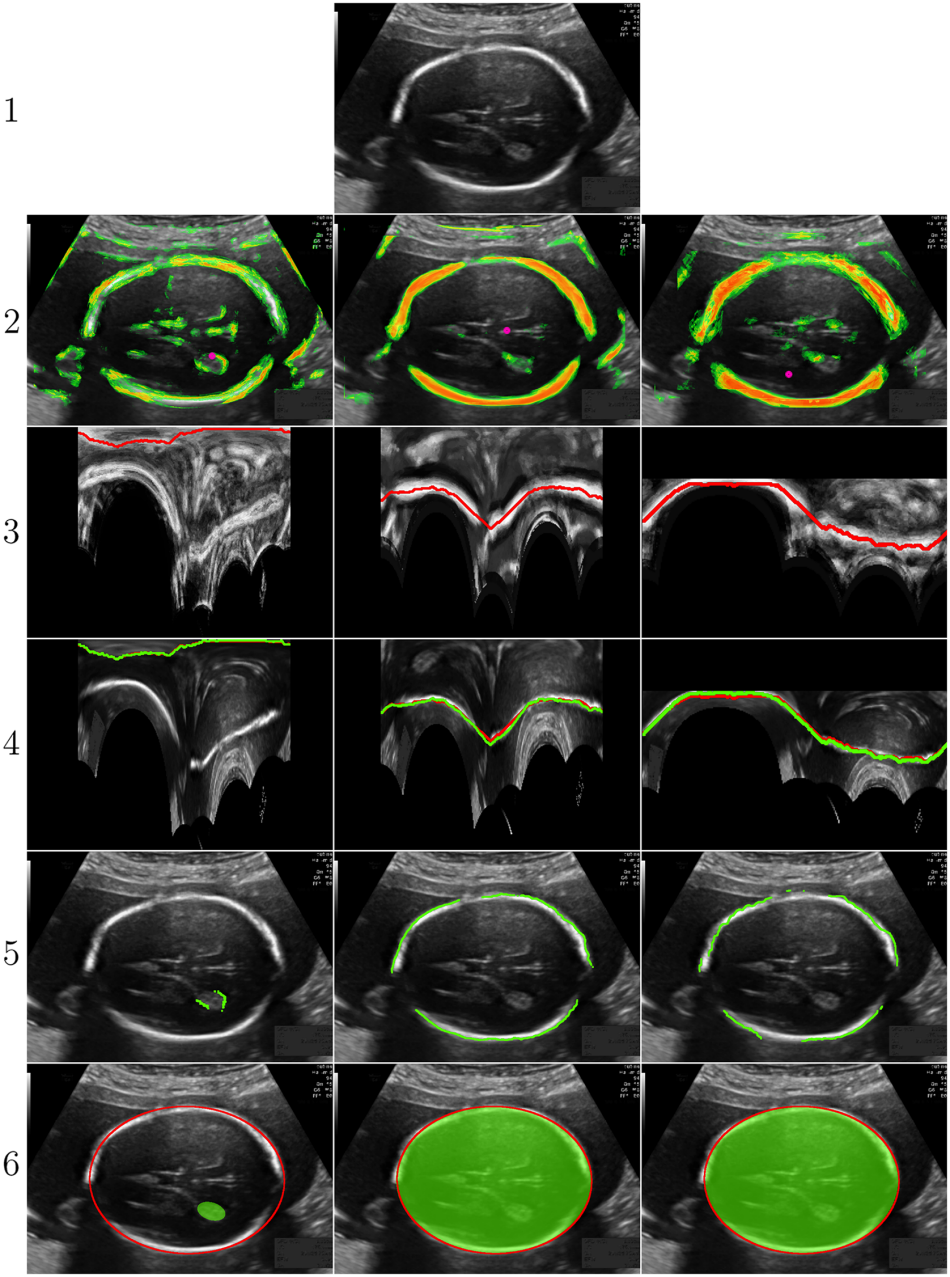
Steps of quantification system *C*. From left to right: pipeline 1, 2, and 3, respectively. From top to bottom: (1) Input image. (2) Input ultrasound image with overlay of pixel classifier likelihood ranging from green to red and Hough transform result in pink. (3) Polar transformed pixel classifier likelihood with overlay of dynamic programming in red. (4) Polar transformed ultrasound image with overlay of dynamic programming in red and repositioned dynamic programming result in green. (5) Ultrasound image with overlay of the highest five percentile repositioned dynamic programming pixels. (6) Ultrasound image with fitted ellipse in green and annotation of the experienced sonographer in red. In this example image, the pipeline that was optimized for the second trimester is automatically selected as the best result, since the edge of this fitted ellipse has the highest median pixel classifier output.

Fig 6 shows the output of each step in quantification system *C* for an ultrasound image in the test set of a fetus with a GA of 20^+0^ weeks. All three pipelines of system *C* are computed on the input image from the test set. The second row shows the output of the pixel classifiers for each pipeline. It can be seen that the pixel classifier of the first pipeline, which is optimized on the training data of the first trimester, does not give a high response on this image. The third row shows the polar transform of the pixel classifier, where it can be seen that the radial dimension of the image decreases as the trimester increases due to the increase in sampling distance *S*_*dis*_. The middle image of the fourth row shows that the second dynamic programming result (green) is re-positioned towards the outside of the fetal skull compared to the first dynamic programming result (red). Row six shows the final three fitted ellipses. In this example, the pipeline that was optimized on the training data of the second trimester gave the highest median pixel classifier response on the edge of the fitted ellipse. This ellipse was therefore selected as the final result.

### HC comparison

Table 4 shows the DF, ADF, HD and DSC of the measured HC from observer 1 compared to quantification systems *A*, *B* and *C* and observer 2. The ADF, HD and DSC of system *A* are significantly worse in the first trimester than systems *B* and *C* and observer 2. In the second trimester, system *A* fails on one image because the system fits an ellipse smaller than 38.6 mm. Therefore, the values for system *A* in the second trimester only consist of 232 values. The DF and ADF of observer 2 are significantly worse in the second trimester than systems *A*, *B* and *C*. There are no significant differences in HC between the systems and observer 2 in the third trimester.

**Table 4:** Results of the experienced sonographer (observer 1) compared to the classifier *A*, *B* and *C* and the medical researcher (observer 2) on the test set.

		Trimester 1	Trimester 2	Trimester 3
DF(mm)	Observer 2	1.4±1.9^•^°	3.4±2.8*^•^°	1.8±7.0
	System A	3.2±20.5	1.1±3.0^•^°	0.7±6.3
	System B	-0.3±6.0	0.9±3.8	1.3±6.7
	System C	-0.3±6.1	0.8±3.3	0.6±5.9
ADF(mm)	Observer 2	1.8±1.5	3.7±2.5*^•^°	5.4±4.6
	System A	11.3±17.3^‡^^•^°	2.3±2.2	5.1±3.7
	System B	3.1±5.1	2.5±3.0	5.4±4.0
	System C	3.1±5.2	2.4±2.4	4.8±3.4
HD (mm)	Observer 2	0.9±0.5	1.8±0.9	3.3±1.6
	System A	5.0±5.7^‡^^•^°	1.8±1.1	3.5±1.6
	System B	1.7±2.3^‡^	1.8±1.4	3.9±2.3
	System C	1.7±2.3^‡^	1.8±1.3	3.3±1.6
DSC(%)	Observer 2	96.8±1.7	97.5±1.0*^•^	97.4±1.0
	System A	84.1±15.2^‡^^•^°	97.6±1.3	97.3±1.1
	System B	94.4±5.4^‡^	97.6±1.5	96.9±1.5
	System C	94.4±5.5^‡^	97.6±1.4	97.2±1.2

Note:

^‡^significantly different from observer 2,

*significantly different from system *A*,

^•^significantly different from system *B*,

°significantly different from system *C*

### GA comparison

**Fig 7:**
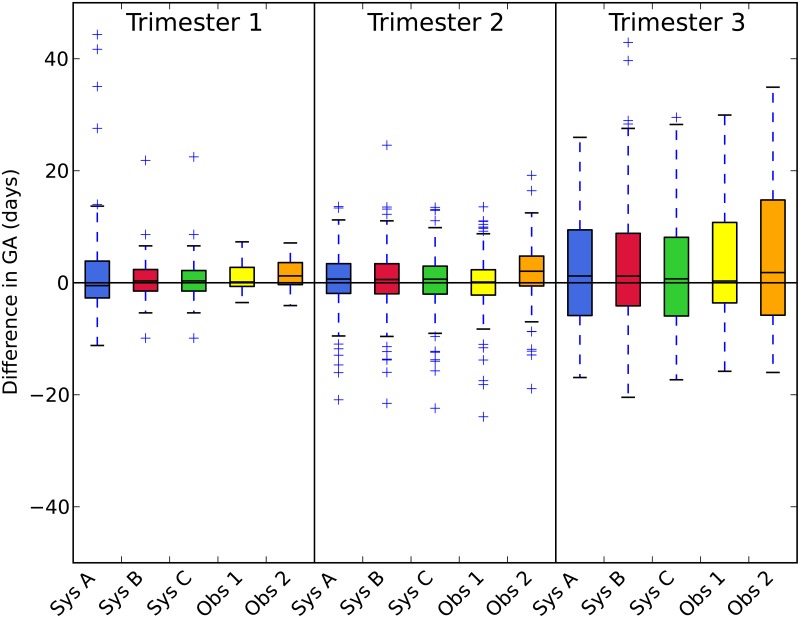
The difference with the reference GA (days) that was estimated using the CRL in the first trimester.

The difference between the reference GA (estimated from the CRL) and the GA computed from the HC is shown in Table 5 and visualized in Fig 7. The difference between the reference and observer 2 in the first trimester is significantly worse than the difference between the reference and observer 1, system *B* and system *C*. The difference between the reference and observer 2 in the second trimester is significantly worse compared to observer 1 and systems *A*, *B* and *C*. Fig 7 shows that observer 2 tended to manually annotate the HC a few millimeters larger compared to observer 1, which resulted in a larger estimated GA. Fig 7 shows that system *A* has a large interquartile range and four outliers with a difference of more than 20 days. System *B* is significantly worse than that of system *C* in the third trimester. This is caused by two outliers with a difference of more than 30 days, which are shown in Fig 7.

**Table 5:** Mean difference with the reference GA (days) that was estimated using the CRL in the first trimester.

	Trimester 1	Trimester 2	Trimester 3
Observer 1	0.8±2.6	-0.0±4.6	1.9±11.0
Observer 2	1.6±2.7^†^^•^°	2.0±4.8*^•^°	3.9±13.7
System A	2.5±11.2	0.6±4.7^†^^•^°	2.9±12.5
System B	0.6±4.3	0.6±4.9^†^	3.8±14.4°
System C	0.6±4.3	0.4±4.7^†^	2.5±12.4

Note:

^†^significantly different from observer 1,

*significantly different from system *A*,

^•^significantly different from system *B*,

°significantly different from system *C*

### Visual results of quantification system C

**Fig 8:**
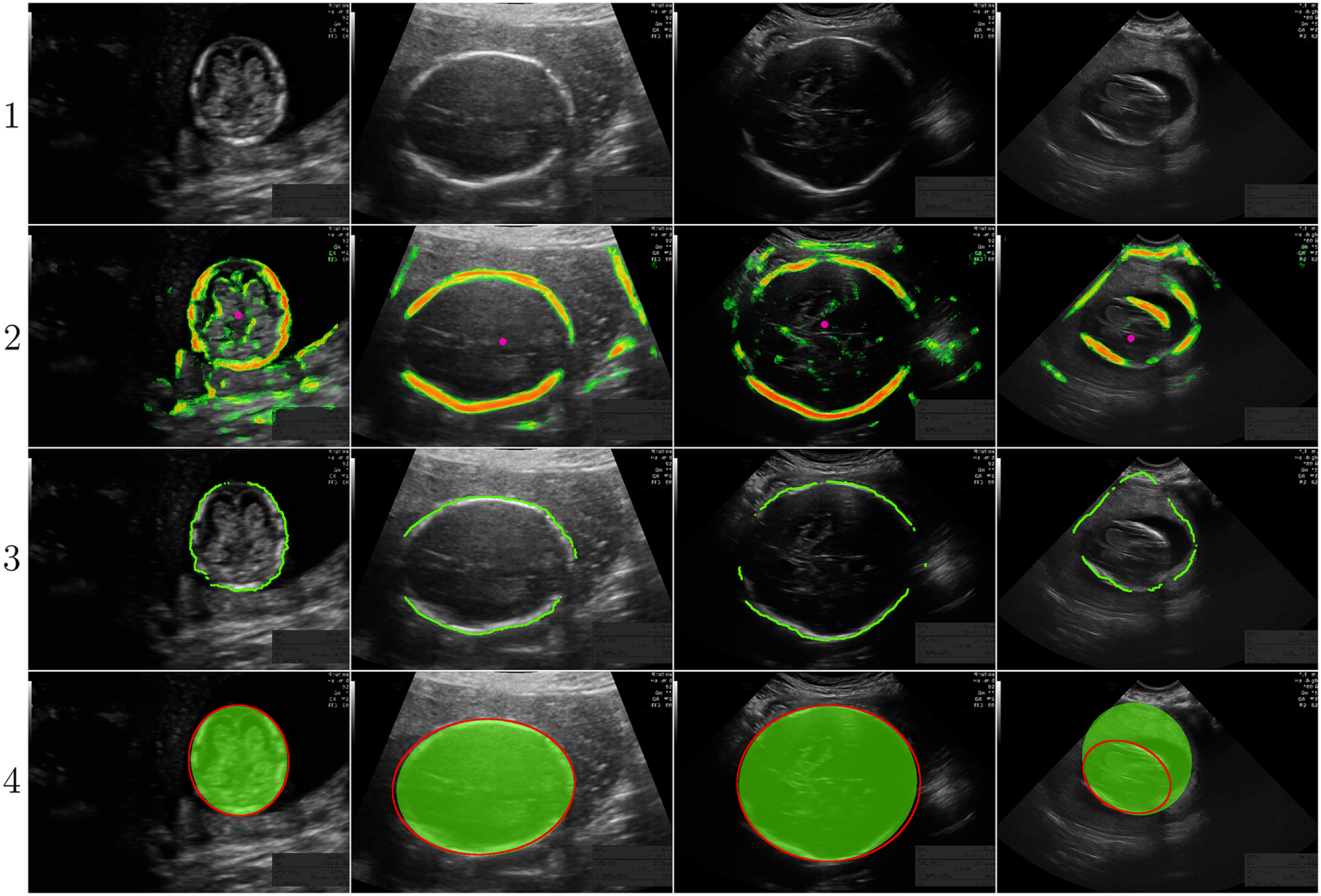
Results of quantification system *C* closest to the median ADF of system *C*. From left to right: first trimester with a ADF of 1.8 mm, result second trimester with a ADF of 1.6 mm, result third trimester with an ADF of 4.2 mm and worst result first trimester with an ADF of 36.8 mm. From top to bottom: (1) The ultrasound image. (2) The ultrasound image with overlay of the pixel classifier likelihood ranging from green to red and the Hough transform result in pink. (3) The ultrasound image with an overlay of the highest fifth percentile repositioned dynamic programming pixels. (4) The ultrasound image with the fitted ellipse in green and the annotation of observer 1 in red.

To get an idea how the median ADF of system *C* looks like, the result closest to the median ADF of system *C* is visualized in Fig 8. The images of the first, second and third trimester have an ADF of 1.8 mm, 1.6 mm and 4.2 mm, which results in a difference in GA of -1.0 days, -0.9 days and -4.3 days, respectively. The median ADF in the first trimester is a lot smaller compared to the mean ADF of 3.1 mm (shown in Table 4), due to one outlier. This outlier is shown in the right column of Fig 8 and has a ADF of 36.8 mm, which results in a difference in GA of 22.5 days with the GA estimated from the CRL.

### Overfitting

Table 6 shows the results of quantification system *C* on the training and the test set. Overfitting occurs when the results on the training set are much better than the results on the test set.

**Table 6:** Results of quantification system C for training and test set compared to observer 1.

	Trimester 1		Trimester 2		Trimester 3	
	Train set	Test set	Train set	Test set	Train set	Test set
DF (mm)	-0.7±6.4	-0.3±6.1	0.7±2.1	0.8±3.3	1.0±6.1	0.6±5.9
ADF (mm)	3.4±5.5	3.1±5.2	2.3±2.1	2.4±2.4	4.6±4.1	4.8±3.4
HD (mm)	2.1±2.7	1.7±2.3	1.7±0.9	1.8±1.3	3.4±2.0	3.1±1.9
DSC (%)	93.2±7.5*	94.4±5.5	97.6±1.8	97.6±1.4	97.2±1.5	97.3±1.5

Note:

*p<0.05

### Comparison to literature

Table 7 shows a comparison of system *C* with the reported results published in literature.

**Table 7:** Comparison of system *C* against the reported results published in literature.

Method	No.	GA (weeks)	DF (mm)	ADF (mm)	HD (mm)	DSC (%)
Our method	335	11-37	0.6±4.3	2.8±3.3	2.0±1.6	97.0±2.8
Zhang *et al*. [17]	10	-	-0.22±9.53	-	3.30±1.09	-
Anto *et al*. [16]	50	-	-	-	-	75±-
Perez-Glez. *et al*. [15]	10	-	-2.73±2.04	-	2.64±0.57	97.19±0.97
Jatmika *et al*. [9]	100	-	-	8.21±-	-	-
Satwika *et al*. [5]	72	Trim 1&2	-	14.6±-	-	-
Foi *et al*. [26]	90	21,28,33	-2.01±3.29	-	2.16±1.44	97.80±1.04
Ciurte *et al*. [26]	90	21,28,33	11.93±5.32	-	4.6±1.64	94.45±1.57
Stebbing *et al*. [26]	90	21,28,33	-3.46±4.06	-	2.59±1.14	97.23±0.77
Sun *et al*. [26]	90	21,28,33	3.83±5.66	-	3.02±1.55	96.97±1.07
Ponomarev *et al*. [26]	90	21,28,33	16.39±24.88	-	6.87±9.82	92.53±10.22
Ni *et al*. [8]	175	17-38	-	5.58±1.74%	-	-
Zalud *et al*. [7]	80	-	-	5.1±5.4	-	-
Carneiro *et al*. [6]	20	-	-	2.76±1.40	4.15±2.05	-
Lu and Tan [4]	11	13-34	-	3.41±1.74%	-	-

## Discussion

We presented three variations of a quantification system, indicated as system *A*, *B* or *C*, that measures the fetal HC in all trimesters of the pregnancy. The systems were evaluated on a large test set of 335 ultrasound images. The best system, system *C*, performs comparable to an experienced sonographer (observer 1) and significantly better than a medical researcher (observer 2) in the first and second trimester. The presented system shows similar or superior results compared to other systems published in literature. This is the first system in literature that was evaluated on a very larger test set of 335 ultrasound images which contained data of all trimesters of the pregnancy (Table 7). In the next Section, we discuss various aspects of the system and the experimental results.

### HC comparison

Table 4 shows that system *A* performs significantly worse in the first trimester compared to systems *B* and *C*. This results from the fact that the appearance of the fetal skull in the first trimester differs from that in the second and third trimester. Since the fetal skull is relatively soft in the first trimester, it does not always appear brighter than the inside of the fetal head. Therefore, it is sometimes very difficult to detect the edge of the fetal head, especially when it lies close to the wall of the uterus. Thus, it is more difficult to automatically measure the HC in the first trimester. For this reason, it is important to show the performance of an automated system for each trimester separately. It is also known that the standard deviation in HC increases as the size of the fetus increases. The primary reason for this results from the fact that the natural variation in fetal size increases with GA. Since the fetal head becomes larger with GA, the pixel size of the ultrasound image also increases. This can also be noticed in the curve of Verburg *et al*., where the P3-P97 interval gets wider with increasing GA. Separation of the different trimesters is hereby essential when evaluating the results. It also underlines the clinical importance to estimate the GA of the fetus in the first or second trimester to obtain a reliable estimate of the fetal GA.

### GA comparison

Table 5 shows that system *B* performs significantly worse than system *C* in the third trimester, so it is beneficial to train a separate classifier for the third trimester as well. Together with the results from the previous Section, it can be concluded that system *C* performs superior to systems *A* and *B* and was therefore chosen as the final system.

Table 5 and Fig 7 show that the standard deviation of the GA in the first trimester of quantification system *C* is larger than the that of the two observers. This is mainly caused by one outlier. When this outlier was removed, the standard deviation decreases from 4.3 to 3.1 days, which is similar to the standard deviation of observers 1 and 2.

The mean GA estimation of system *C* is significantly better than observer 2 in the first and second trimesters, compared to the reference GA estimated from the CRL. The underlying reason for this is that observer 2 systematically annotated the HC a few millimeters larger compared to observer 1. This indicates that the system may aid inexperienced human observers in measuring the HC. Furthermore, the standard deviation of the HC in the second trimester is similar for both observers and system *C*. The performance of observer 1 is significantly better in the second trimester compared to system *C*, but the mean difference of 0.4 days is not clinically relevant.

### Visual results of quantification system C

Fig 8 shows the result of system *C* with the median ADF for each trimester. It can be seen that the median result of system *C* is very similar to the manual annotations of observer 1. The increase in ADF for later trimesters is mainly caused by the increase in pixel size. The right column in Fig 8 shows the outlier of system *C* in the first trimester. In this image, the right and left side of the fetal skull are hardly visible. In addition, a large shadow appears next to the dark amniotic fluid at the right side of the fetal skull. While the Hough transform still detects the center of the fetal head, the dynamic programming algorithm is not able to follow the fetal skull. Instead, it follows the border between the amniotic fluid and the shadow, resulting in a HC that is completely off. This results in a difference in GA of 22.5 days with the reference GA.

### Overfitting

Table 6 shows the results of system *C* on the training and the test sets. Note that no overfitting occurs because the results from the training and test sets did not differ significantly. The DSC in the first trimester was even significantly worse in the training set compared to the test set.

### Comparison to literature

Table 7 shows an overview of previously reported results in literature. Ideally, these methods were evaluated on the same test set to make a direct comparison possible. Unfortunately, such a dataset was not available and implementation of other methods is a difficult task due to the lack of implementation details. Even though a direct comparison of the results is not possible, Table 7 highlights three strengths of our method. First, four methods [4, 6, 15, 17] were only evaluated on a dataset of 10, 11 or 20 images. Our method was evaluated on a large independent test set of 335 images, which shows not only the feasibility but also the robustness of the method. Secondly, it was shown that the first trimester is the most challenging trimester to measure the HC, but almost all other methods either did not mention the GA of the test set, or only evaluated their system only on data of the second and third trimester. We therefore recommend that future research will report the GA and evaluate the results for each trimester separately. This would make a comparison with previous work easier. Thirdly, only Satwika *et al*. [5] have evaluated their system on a relatively large test set of 72 images which included data of the first trimester. They have reported a mean ADF of 14.6 mm, which is much larger compared to the ADF of 2.8±3.3 mm of our proposed method. Even though these systems were not evaluated on the same test set, it illustrates the potential of our proposed method.

### Study limitations

The data for this study was acquired in only one hospital using two different ultrasound devices from the same vendor. Future work should include multi-center data from different vendors to be able to further evaluate the performance of the proposed method. The results show that the system performs significantly better than a medical researcher in the first and second trimester, but it is still required to obtain the 2D standard plane. Other work in literature focuses on aiding less skilled sonographers in obtaining the 2D standard plane, or reconstructing the 2D standard plane from a 3D volume [27–32]. Combining these methods with our proposed system could further improve inter-observer variability, but this is out of the scope of this work.

## Conclusions

We presented an automated system for the detection of fetal HC in 2D ultrasound images. This is the first system presented in literature that was evaluated on a large independent test set of 335 ultrasound images that included data of all trimesters. It was shown that it is important to separate the results for each trimester, because the uncertainty of the estimated GA increases with GA due fact that the natural variation in fetal size increases with GA. This is the first system that evaluated results for each trimester separately. The GA can be estimated more accurately in the first trimester, but the fetal skull is not clearly visible in the first trimester, which makes automated detection of the HC a more challenging task. The performance of the presented system was comparable to an experienced sonographer.

## Supporting information

S1 File (XLSX)

S2 File (XLSX)
